# Assessing the impact of resistance training on renal function of female wistar rats under cross-sex hormone therapy

**DOI:** 10.3389/fphys.2025.1543077

**Published:** 2025-05-26

**Authors:** Isadora Gonçalves Almeida, Isabela Borges M. Silveira, Emily Rocha Cordeiro, Letícia Maria Monteiro, Nathalia Beserra da Silva, Rogerio Argeri, Debora C. K. Lichtenecker, Magnus R. Dias da Silva, Guiomar Nascimento Gomes

**Affiliations:** ^1^ Laboratory of Renal Physiology Department of Physiology, Escola Paulista de Medicina, Universidade Federal de São Paulo (EPM/Unifesp), São Paulo, Brazil; ^2^ Postgraduate Program in Translational Medicine, Department of Medicine, Escola Paulista de Medicina, Federal University of São Paulo, São Paulo, Brazil; ^3^ Laboratory of Molecular and Translational Endocrinology (LEMT), Endocrinology Division, Department of Medicine, Escola Paulista de Medicina, Universidade Federal de São Paulo (EPM/Unifesp), São Paulo, Brazil; ^4^ Trans Care Outpatient Clinics; Núcleo de Estudos, Pesquisa, Extensão e Assistência à Pessoa Trans Professor Roberto Farina, Universidade Federal de São Paulo (Núcleo TransUnifesp), São Paulo, Brazil

**Keywords:** cross-hormone-therapy, testosterone, physical exercise (EX), resistance training, renal function

## Abstract

**Introduction:**

Cross-hormone therapy (CHT) is commonly used in the gender-affirming process, with testosterone being administered to trans men to develop secondary masculine characteristics. In experimental models replicating this condition, CHT has been associated with increased plasma creatinine levels and renal morphological changes. Given benefits of physical exercise, this study aimed to evaluate whether resistance training could mitigate CHT-induced renal alterations.

**Objectives:**

To investigate the impact of resistance training combined with CHT on blood pressure and renal morphology and function.

**Methods:**

Two-month-old female Wistar rats were divided into four groups: FSV–sedentary rats treated with vehicle (vegetable oil); FSH–sedentary rats treated with CHT; FEV–exercised rats treated with vehicle; and FEH–exercised rats treated with CHT. CHT was administered via testosterone cypionate (3.0 mg/kg, intramuscularly) every 10 days for 8 weeks. Exercise groups underwent progressive resistance training using a vertical climbing ladder five times per week for 6 weeks. At the end of the protocol, the animals were placed in metabolic cages for urine collection, followed by blood sampling for biochemical analysis.

**Results:**

Testosterone-treated groups showed increased plasma creatinine levels, though urea concentrations were unchanged. Plasma sodium concentration was elevated, and sodium excretion was reduced in the sedentary testosterone-treated group. Morphological analysis revealed that resistance exercise reduced macrophage infiltration, lowered the number of PCNA-positive cells in kidney tissue, and decreased glomerular tubularization in the kidney.

**Conclusion:**

Testosterone-based CHT in female rats induces renal functional alterations, but resistance exercise effectively attenuates these effects by reducing macrophage infiltration, cell proliferation, and glomerular changes.

## 1 Introduction

Sex steroids play a crucial role in the gender-affirming process, with cross-sex hormone therapy (CHT) serving as a key component. CHT involves the administration of exogenous steroid hormones, often beyond endogenous production levels. Testosterone is commonly prescribed to transgender men to suppress estradiol and other female-associated hormones, facilitating the development of masculine secondary sexual characteristics (Figueiredo et al., 2022; Irwig, 2017). However, testosterone use has been linked to potential physiological consequences, including alterations in cardiovascular, hepatic, and endocrine function (Carmo et al., 2011; Abrahin et al., 2013).

The effects of testosterone on kidney function remain a subject of debate, as the hormone exerts complex influences on renal vasculature. In glomerular capillaries, testosterone appears to reduce the vascular tone of afferent arterioles (Lu et al., 2012) while in other regions, increases sensitivity to vasoconstrictive agents (Baker et al., 1978). It also modulates the thromboxane/prostaglandin balance (Wakasugi et al., 1989), inhibits nitric oxide (NO) synthesis, and promotes pro-inflammatory responses (Park et al., 2004). In the context of renal ischemia and reperfusion (I/R), testosterone has been associated with exacerbated kidney injury (Park et al., 2004). However, in male patients with hypogonadism and end-stage renal disease, testosterone replacement therapy has demonstrated beneficial effects (Snyder and Shoskes, 2016). Despite these contrasting findings, both clinical and experimental studies suggest that testosterone use in CHT may predispose individuals to renal alterations, including elevated plasma creatinine levels and glomerular morphological changes (Lichtenecker et al., 2021; Turino Miranda et al., 2024; Krupka et al., 2022).

The health benefits of physical activity, particularly resistance training, are well-documented. Resistance training is widely recommended for maintaining overall health in the general population (Fisher et al., 2011; Ribeiro et al., 2014; Palop Montoro et al., 2015) and plays a crucial role in the prevention and management of chronic diseases (Kohl et al., 2012). For example, in individuals with type 1 diabetes, resistance training has been shown to reduce the risk of microalbuminuria and slow the progression of kidney disease (Wadén et al., 2015).

Chronic kidney disease represents a growing global health concern, with significant economic and social implications (Francis et al., 2024). In transgender individuals, prolonged exposure to exogenous sex steroids can lead to significant physiological changes in renal function and morphology. Given these potential effects, investigating the interplay between resistance training and CHT in experimental models is crucial to determining whether exercise can mitigate the adverse renal consequences of hormone therapy.

## 2 Objectives

The aim of this study is to evaluate the impact of a resistance exercise program associated with cross-hormonalization on the renal function and morphology of female rats.

## 3 Methods

### 3.1 Experimental design

Two-month-old female Wistar-EPM rats were purchased from the Center for the Development of Experimental Models for Medicine and Biology (CEDEME/UNIFESP). All experimental procedures were approved by the University Ethics Committee (protocol 9009241022). The animals had free access to standard laboratory chow and water throughout the experimental protocol and were housed in ventilated cages, in groups of 2–3 animals, in a room at constant temperature (22°C) under a 12-h light/dark cycle (lights on at 7 a.m.).

**FIGURE 1:**
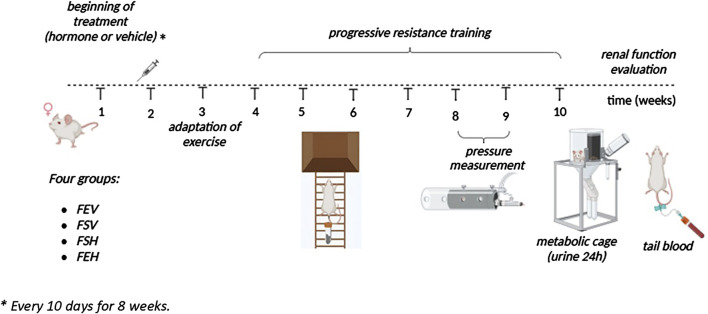
Schematic representation of the experimental design.

The female rats were divided into four experimental groups: female sedentary oil (FSO), female sedentary hormone (FSH), female exercise oil (FEO) and female exercise hormone (FEH). The FSH and FEH groups received testosterone cypionate (3.0 mg/kg, i.m.), while the FSO and FEO groups received sesame oil as a vehicle every 10 days for 8 weeks. The animals in the FEO and FEH groups underwent a progressive resistance training program. The dose of the hormone was based on the study of Lichtenecker et al. (2021). The schematic representation of the experimental design is shown in Figure 1.

### 3.2 Resistance exercise training protocol

Initially, the animals were familiarized with the climbing apparatus, consisting of an 80° inclined vertical ladder apparatus (110 cm high × 18 cm wide, with 2-cm grid steps). A housing chamber (L × W × H = 20 × 20 × 20 cm) was located at the top of the ladder and served as a shelter during the resting period. Familiarization took place over 3 days, with three attempts each day. Initially, the animal was placed in the shelter for 60 s, and then the trials were carried out (adapted from Cassilhas et al. (2013)). After the adaptation period, the animals in the exercise groups underwent a maximum load test, starting with a load equivalent to 75% of their body mass. If they complete the climb without difficulty, the load is progressively increased to 90%, 100%, or more, until the animal can no longer ascend (from 100% of body weight onward, additional loads of 30 g are added per climb). The last successfully completed load is recorded as the maximum load.

The animals underwent a progressive resistance training protocol five times per week for 6 weeks. Each session consisted of eight climbing sets with a load attached to the tail. The load started at 50% of the maximum load in the first two sets, increasing to 75% (third and fourth sets), 90% (fifth and sixth sets), and 100% (seventh and eighth sets). During training, the animals must climb the ladder spontaneously. The rest interval between sets was 60 s, and each session lasted between 20 and 30 min.

Weekly, the maximum load test was repeated to adjust the individual overload. At the end of 6 weeks, the maximum load carried was 689.7 g (247.0% of body weight) for the FEH group and 661.0 g (262.4% of body weight) for the FEO group.

### 3.3 Assessment of systolic blood pressure

The caudal blood pressure was measured at 4 months of age using the indirect tail plethysmography technique as described previously (Lichtenecker et al., 2021; Lima et al., 2014). After the adaptation period, five measurements were taken in sequence, for each rat, and the caudal pressure value was obtained by the average of these measurements and expressed in mmHg (Lima et al., 2014).

### 3.4 Renal function parameters

Renal function was assessed at 4 months of age. After the end of the training period, the animals rested for 2–4 days and we started the evaluation of renal function. The rats were placed in metabolic cages for 24 h, when urine and blood samples were collected to determine creatinine, sodium and potassium concentrations. Creatinine levels in plasma and urine were measured using the Jaffé method and the creatinine clearance was determined. Sodium and potassium concentrations were measured by flame photometry (Analyser 910). Plasma urea was determined using an enzymatic method (Labtest Diagnóstica). Arterial pH, HCO3, pCO2 and pO2 were determined in a whole blood sample with an i-STAT Clinical Analyzer (Abbott Point of Care Inc.).

For inulin and para-amino hippurate (PAH) clearance evaluation, the rats were anesthetized with sodium thiopental (60 mg/kg i.p.); the trachea was assessed to maintain ventilation. The right carotid artery, the bladder, and the external jugular artery of each animal were assessed for blood and urine sampling or for infusion of solutions. The glomerular filtration rate (GFR) and renal plasma flow (RPF) were evaluated by clearance of the respective substances (inulin and PAH). Urinary flow values were normalized per kg. Colorimetric methods were used to determine inulin and PAH concentrations (Nascimento-Gomes et al., 1997).

### 3.5 Morphological analysis

The kidneys were weighed and fixed in Bouin and embedded in paraffin for morphological assessment. Histological sections (5 μm thick) were stained with hematoxylin and eosin. Images were acquired (×200 magnification) on a microscope (Nikon H550L) connected to a microcomputer by a video camera (Sony-CCD-IRIS). The analysis of glomerular tubularization was carried out, as established by Simon et al. (2011), which consists of evaluating approximately 100 glomeruli from each animal, recording how many of them have, inside the Bowman’s capsule, proximal convoluted tubule characteristic cells. During this evaluation, the glomeruli can be classified into three grades according to the number of these cells within the glomerulus as presented by Silva et al. (2025) and shown in the adapted scheme found in Supplementary data. From the total of glomeruli analyzed the amount that had tubularization is presented as the percentage.

Immunohistochemistry assays were used to identify ED1-positive cells (macrophages/monocytes), PCNA (Proliferating Cell Nuclear Antigen) and αSMA (Alpha Smooth Muscle Actin). For this purpose, the tissue sections were incubated for 12 h at 4°C with a monoclonal anti-ED1, anti-PCNA and anti-αSMA antibody (1:500 Serotec; 1:300 Sigma-Aldrich; 1:1,000 Dako). The reaction product was determined using an immunoperoxidase polymer (Histofine, Nichirei Biosciences Inc.). After the reaction, the slides were dehydrated and mounted. For quantitative analysis, the percentage of the area or positive cell number was assessed in 20 consecutive fields for each sample (×200 magnification); each field had an area of 275,000 μm^2^.

### 3.6 Statistical analysis

All statistical analyses were performed using GraphPad Prism 6.0 (GraphPad Software Inc.). The normality assumption was based on Shapiro-Wilk’s W test. The results expressed as mean ± standard error (SEM) and were analyzed by Two-Way ANOVA followed by Tukey. The level of significance was set at 5% (p ≤ 0.05). Effect sizes were reported in the Figures as eta squared (ƞ2). As part of two-way ANOVA, Prism reports the % of total variation accounted for by the interaction, the column factor and the row factor. These values are computed by dividing the sum-of-squares from the ANOVA table by the total sum-of-squares and these values (% of total variation) are called standard omega squared by Sheskin (Equations 27.51 - 27.53, and R2 by Maxwell and Delaney (page 295). Others call eta squared (ƞ^2^) or the correlation ratio (Lakens, 2013).

Considering that Principal Component Analysis (PCA) can assist in identifying patterns and underlying relationships among renal function, inflammatory markers, and histological alterations, we performed this analysis as a complement to the statistical tests used. The results are presented in the form of a biplot with clusters (Supplementary Figure 2). Cluster overlay was also performed using the *k*-means method (Supplementary Figure 3). These analyses were conducted following the approaches described by Wakeling and MacFie (1995), Filzmoser et al. (2009) and Kassambara et al. (2020).

## 4 Results

### 4.1 Anthropometric and functional parameters changes

Table 1 presents the anthropometric and functional parameters of the studied groups. Cross-hormone therapy (CHT) significantly increased body weight in both sedentary and exercised rats, and this was mirrored by an increase in nasoanal length. Additionally, CHT resulted in a notable reduction in uterine weight, likely due to testosterone’s inhibitory effects on endogenous female hormone production. Interestingly, a reduction in uterine weight was also observed in the group subjected solely to resistance training, suggesting that the applied physical training was of high intensity as stated by Pardini (2001).

**TABLE 1:** Summary of urinary and blood parameters observed in 4-month-year-old female rats submitted to CHT and/or Resistance exercise training protocol.

Parameter	Sedentary		Exercise		Two-way-ANOVA		
	Control (oil) (N = 13)	Hormone (N = 12)	Control (oil) (N = 16)	Hormone (N = 12)	Interaction effect	Exercise effect	Hormone effect
Body weight (g)	244.7 ± 5.60	281.2 ± 9.08	251.4 ± 7.83	273.5 ± 4.95	p = 0.2983	p = 0.9736	**p = 0.0274**
Nasoanal length (cm)	22.05 ± 0.23	23.35 ± 0.46*	22.40 ± 0.32	22.63 ± 0.15	p = 0.1032	p = 0.5695	**p = 0.0234**
Lee index (g^1/3^/cm)	283.7 ± 2.11	280.8 ± 3.51	281.8 ± 3.13	286.9 ± 1.75	p = 0.1629	p = 0.4684	p = 0.6978
Kidney weight (g)	2.62 ± 0.19	2.55 ± 0.11	2.78 ± 0.07	2.57 ± 0.07	p = 0.4523	p = 0.4130	p = 0.0778
Ovaries and uterus weight (g)	1.11 ± 0.06	0.67 ± 0.10*	0.96 ± 0.06	0.86 ± 0.05	**p = 0.0436**	p = 0.8756	**p = 0.0169**
Ovaries and uterus (% body weight)	0.4530 ± 0.019	0.236 ± 0.034*	0.416 ± 0.033^#^	0.338 ± 0.028	**p < 0.001**	**p < 0.001**	**p < 0.001**
Blood Pressure (mmHg)	119.1 ± 1.26	117.6 ± 4.16	119.5 ± 3.41	128.2 ± 3.88	p = 0.3204	p = 0.3209	p = 0.8702
Blood							
pH	7.36 ± 0.012	7.38 ± 0.014	7.33 ± 0.015	7.35 ± 0.013	p = 0.8632	**p = 0.0388**	p = 0.2755
pCO_2_ (mmHg)	46.20 ± 1.33	45.88 ± 3.04	53.51 ± 3.23	50.01 ± 2.59	p = 0.5402	**p = 0.0336**	p = 0.4613
HCO_3_^−^ (mmol/L)	26.13 ± 0.90	26.58 ± 0.88	28.17 ± 1.18	27.14 ± 0.61	p = 0.4202	p = 0.1605	p = 0.7451
Hematocrit (%PCV)	41.11 ± 1.77	42.50 ± 1.16	40.29 ± 0.94	42.25 ± 0.59	p = 0.8228	p = 0.6758	p = 0.1983
Creatinine (mg/dL)	244.7 ± 5.60	281.2 ± 9.08*	251.4 ± 7.81^#^	273.5 ± 4.95*^#^	p = 0.6135	**p < 0.0001**	**p = 0.0001**
Urea (mg/dL)	35.44 ± 1.67	36.79 ± 1.47	38.13 ± 0.99	39.50 ± 1.77	p = 0.9940	p = 0.0962	p = 0.3932
[Na^+^]_p_ (mmol/L)	148.2 ± 1.21	154.7 ± 0.97*	143.9 ± 0.59^#^	145.6 ± 0.96*	**p = 0.0335**	**p < 0.0001**	**p = 0.0005**
[K^+^]_p_ (mmol/L)	3.86 ± 0.085	4.14 ± 0.145	3.67 ± 0.131	3.90 ± 0.096	p = 0.6799	p = 0.1457	p = 0.0646
Urinary							
Urinary flow (mL/min/kg)	0.032 ± 0.001	0.028 ± 0.003	0.032 ± 0.002	0.033 ± 0.002	p = 0.7420	p = 0.8003	p = 0.9791
Na^+^ excretion (mEq/24 h)	4.27 ± 0.22	3.09 ± 0.44	4.06 ± 0.27	3.61 ± 0.56	p = 0.3445	p = 0.6969	**p = 0.0384**
K^+^ excretion (mEq/24 h)	13.02 ± 1.21	8.25 ± 0.61*	9.64 ± 0.54	8.3 ± 0.5	**p = 0.0035**	p = 0.9336	**p = 0.0469**

Differences statistically significant when p < 0.05; vs. control* or sedentary^#^. Tukey post test after two-way-ANOVA., Values are means ± standard error. N = number of measurements. The signifcant values are shown in bold.

Blood pressure values for all groups remained within the normal range; however, the CHT-exercised group showed values approaching the upper limit of normality. CHT also induced significant increases in plasma creatinine (p < 0.0001) and sodium concentrations (p < 0.0005), while simultaneously reducing urinary sodium excretion in CHT groups.

### 4.2 Glomerular and hemodynamic parameters

**FIGURE 2:**
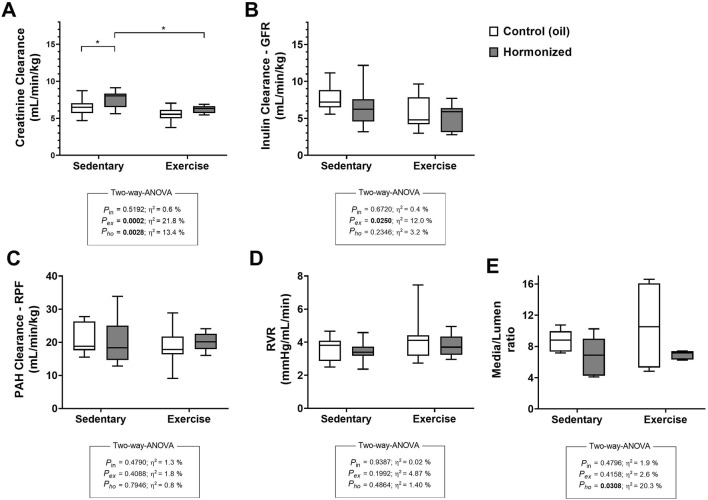
Creatinine **(A)** Inulin **(B)** and PAH **(C)** clearances, renal vascular resistance–RVR **(D)** and media/lumen ratio **(E)** of renal interlobular arteries observed in 4-month-year-old female rats submitted to CHT and/or resistance exercise training protocol. Values are mean ± standard error. Two-way ANOVA followed by Tukey’s post-hoc test, *p ≤ 0.05; **p ≤ 0.01; ****p ≤ 0.001.

Figure 2 illustrates the glomerular and hemodynamic parameters. Creatinine clearance was significantly affected by both CHT and exercise (Figure 2A). In contrast, inulin clearance was influenced solely by exercise (Figure 2B). The media-to-lumen ratio of renal interlobular arteries was significantly reduced by CHT (Figure 2E). However, renal plasma flow, as assessed by PAH clearance, and renal vascular resistance (RVR) were not affected by either CHT or exercise (Figures 2C,D).

### 4.3 Histological analyses

**FIGURE 3:**
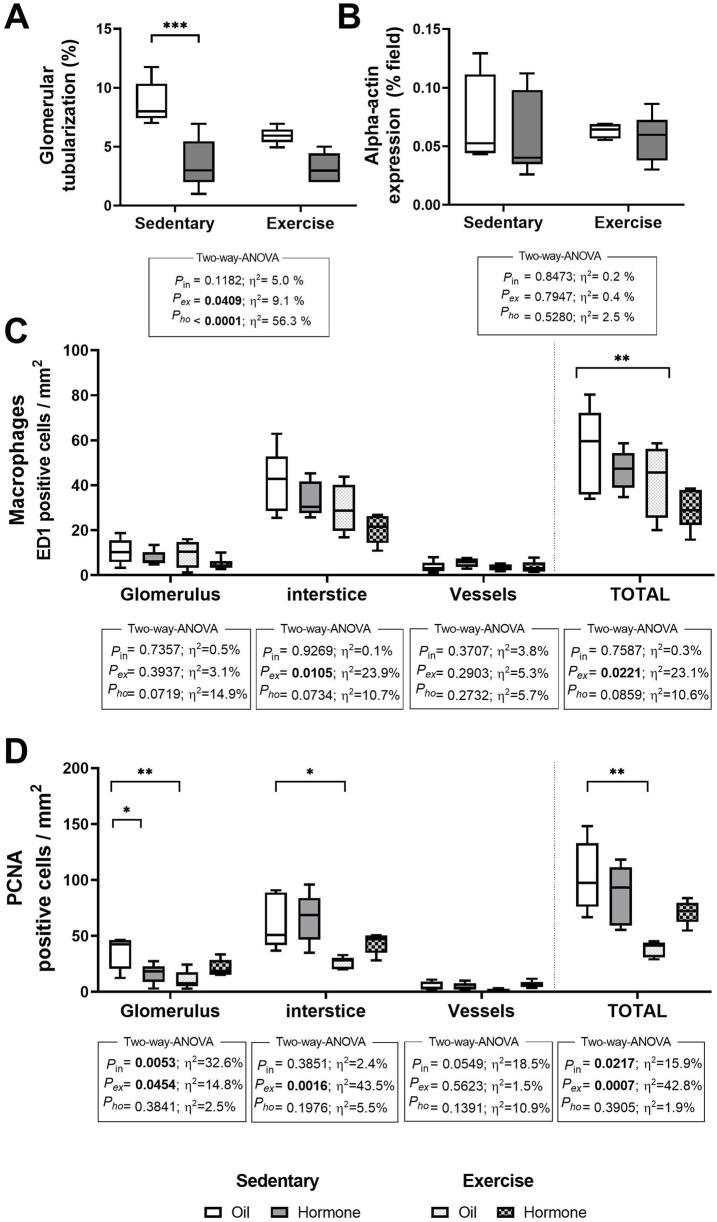
Glomerular tubularization **(A)**, Alpha Smooth Muscle Actin expression **(B)**, ED1 **(C)** and PCNA **(D)** positive cells. Values are mean ± standard error. Two-way ANOVA followed by Tukey’s post-hoc test, *p ≤ 0.05; **p ≤ 0.01; ****p ≤ 0.001.

Figure 3 showe the results of kidney injury markers from histological analysis. Glomerular tubularization was significantly reduced by both CHT and exercise (Figure 3A). Resistance training effectively reduced the total number of macrophages in renal tissue (Figure 3C). A positive impact of exercise was also evident in the reduced number of PCNA-positive cells, indicating lower cellular proliferation in the kidney tissue (Figure 3D). However, neither CHT nor exercise influenced alpha-actin expression levels (Figure 3B).

Overall, CHT induced notable alterations in renal function, including increased plasma creatinine and sodium levels, and reduced urinary sodium excretion; exercise reduced creatinine and inulin clearances. Resistance exercise demonstrated a protective effect by reducing renal injury markers, such as macrophage infiltration and PCNA-positive cells, while also diminishing glomerular tubularization.

## 5 Discussion

This study evaluated renal morphology and function in female Wistar rats subjected to cross-sex hormone therapy (CHT) with or without resistance exercise. We found that CHT significantly increased body weight and nasoanal length, effects that were not modified by physical exercise. Additionally, CHT led to a significant reduction in uterine weight, confirming the suppression of endogenous hormones, as observed in previous studies (Lichtenecker et al., 2021).

### 5.1 Renal function and damage markers

CHT resulted in increased plasma creatinine and sodium concentrations, along with reduced urinary sodium excretion, in both sedentary and exercised animals. Interestingly, resistance exercise modulated markers of kidney damage, reducing macrophage infiltration in kidney tissue, lowering the number of PCNA-positive cells, and decreasing the incidence of glomerular tubularization in all studied groups.

The effects of CHT on body weight and nasoanal length align with the anabolic action of testosterone, which enhances muscle development through increased protein synthesis and stimulation of muscle cell proliferation and differentiation (Kraemer and Ratamess, 2005; Cruzat et al., 2008). The observed rise in plasma creatinine concentration is consistent with findings from Krupka et al. (2022) for transgender individuals undergoing CHT. This increase may be attributed not only to renal dysfunction but also to testosterone’s metabolic effects on muscle, as increased muscle mass can elevate creatinine levels (Calles-Escandon et al., 1984; de Siqueira Guedes et al., 2022).

Interestingly, CHT-S rats exhibited higher creatinine clearance, potentially reflecting the hormone’s effects on tubular creatinine secretion, a phenomenon more pronounced in females (Harvey et al., 1966). However, creatinine clearance in the CHT-E group was significantly lower than in the CHT-S group. To address potential confounders in creatinine clearance, inulin clearance was also assessed, revealing no impact from CHT but a significant effect of physical activity.

The role of sex steroids in renal function is complex, particularly regarding the effects of testosterone on the kidneys (Zhao and Schooling, 2020). Experimental evidence suggests that testosterone may contribute to renal damage, as it can induce death of renal epithelial cells, depending on the dose administered (Peng et al., 2019; Hewitson et al., 2016; Fortepiani et al., 2003). In male rats, castration, in addition to its effects on blood pressure, significantly reduces proteinuria and renal morphological changes (Fortepiani et al., 2003; Baylis, 1994). However, the evidence obtained in studies with humans is limited (Zhao and Schooling, 2020). In a randomized clinical study with a sample size of 48 men, it was shown that the use of testosterone for 6 months significantly decreased the glomerular filtration rate (eGFR) (Pedersen et al., 2017). On the other hand, in patients with CKD, the high testosterone levels have been related to lower rates of death (Khurana et al., 2014) and higher glomerular filtration rate (Kurita et al., 2016).

In a more recent study, the relationship between glomerular filtration rate and serum testosterone levels was evaluated in individuals without kidney disease. In men, higher serum testosterone levels were associated with better kidney function, whereas in women, higher serum testosterone levels were linked to worse kidney function (van der Burgh et al., 2023). To explore the effects of gender-affirming hormone therapy (GAHT) on renal function, van Eeghen et al. (2023) studied changes in serum creatinine and cystatin C levels in individuals undergoing 1 year of GAHT. Their findings indicated that cystatin C-based eGFR increased with estradiol and antiandrogen therapy but decreased with testosterone therapy. Similar results were observed by Turino Miranda et al. (2024). These findings suggest that women with elevated androgen levels may be at a higher risk of developing kidney dysfunction and should be monitored closely to prevent CKD development (van der Burgh et al., 2023).

### 5.2 Exercise-induced sympathetic nerve activity

The intensity, type, and duration of exercise can influence renal function by increasing sympathetic nerve activity to the kidneys (SNAr), leading to a redistribution of blood flow toward active muscles (Rocha et al., 2023; Koçer et al., 2011). Elevated SNAr may impact renal function by enhancing sodium reabsorption and/or reducing the glomerular filtration rate (DiBona, 2005). The high-intensity resistance exercise protocol used in this study may have induced transient vasoconstriction of the afferent arterioles, which could explain its effect on glomerular filtration rate (Rocha et al., 2023). However, despite these physiological responses, no significant changes were observed in renal plasma flow or renal vascular resistance.

### 5.3 Kidney protection by resistance exercise

The scientific literature has debated the participation of inflammatory processes in developing kidney disease and the possible modulation by exercise. It is essential to highlight that performing very intense physical activity can even be associated with acute kidney injury. This condition involves the production of cytokines, activation of immune cells (macrophages, lymphocytes, and neutrophils, among others), and increased oxidative stress. However, moderate exercise may positively impact kidney function. Recently, Costanti-Nascimento et al. (2023) published a review showing that regular and/or moderate exercise could modulate the immune system towards a more regulatory immune response. In this work, the authors found that exercise reduced oxidative stress, inflammatory markers (caspase-3, lactate dehydrogenase, and nitric oxide), the production of inflammatory cytokines (interleukin (IL)-1b, IL-6, IL-8 and also tumor necrosis factor-α (TNF-α)), and tumor necrosis factor-β (TGF-β), a cytokine associated with the development of fibrosis.

In this study, physical exercise demonstrated significant protective effects against kidney damage. Macrophage infiltration, assessed through CD68-positive cell counts, was markedly reduced, in the exercised groups. This aligns with prior findings that resistance training decreases macrophage infiltration and suppresses pro-inflammatory cytokine expression (Bradley et al., 2008; Bruun et al., 2006). Additionally, resistance training promotes systemic anti-inflammatory effects, contributing to improved metabolic health (Arikawa et al., 2011; Balducci et al., 2010; Campbell et al., 2009; Stewart et al., 2007).

Further supporting the protective role of resistance exercise, PCNA expression was significantly reduced in the exercised groups. Since elevated PCNA expression is a marker of cellular proliferation associated with tissue damage and repair processes (Kelman, 1997; Thomas et al., 1998), this finding suggests that resistance exercise helps mitigate kidney injury. Moreover, the combined effects of testosterone and resistance exercise in reducing PCNA-positive cells indicate potential synergistic benefits in protecting renal health (Sinuani et al., 2009).

Glomerular tubularization, a hallmark of early kidney damage (Simon et al., 2011), was also reduced in rats subjected to CHT and resistance exercise. This suggests that physical activity may help preserve renal structural integrity and counteract hormone-induced kidney alterations.

### 5.4 Renal vascular changes

Although CHT altered the media-to-lumen ratio of interlobular arteries, suggesting potential vascular remodeling, this did not result in increased renal vascular resistance. This finding is consistent with Lichtenecker et al. (2021), who reported elevated systolic blood pressure following prolonged hormone therapy without the vascular remodeling typically associated with hypertension. Therefore, the vascular changes observed in this study may reflect early-stage adaptations rather than pathological remodeling.

### 5.5 Clinical significance

In our animal model, resistance training reduced markers of kidney damage in rats undergoing testosterone-based cross-hormone therapy (CHT), including decreased macrophage infiltration, reduced cell proliferation (fewer PCNA-positive cells), and less glomerular tubularization. These results suggest that exercise may help mitigate CHT-related kidney alterations.

However, further research is needed to confirm whether these benefits apply to transgender individuals on testosterone therapy. Human studies are essential to validate these findings and guide tailored exercise protocols.

Our study did not assess the optimal intensity or volume of resistance training for kidney protection. While moderate to vigorous resistance training has shown general cardiovascular and renal benefits, future research should define the specific thresholds needed for protective effects in this context.

Overall, resistance training appears promising for protecting kidney health during testosterone therapy. If confirmed in human studies, it could inform exercise guidelines for transgender individuals, potentially enhancing long-term renal health alongside other lifestyle interventions.

## 6 Conclusion

In summary, CHT in female rats induces alterations in renal function; however, resistance exercise significantly attenuates these effects. This protective influence is evidenced by reduced macrophage infiltration, lower PCNA-positive cell counts, and decreased glomerular tubularization. These findings highlight the potential therapeutic role of physical activity in preserving renal health during testosterone-based hormone therapy.

## Data Availability

The original contributions presented in the study are publicly available. This data can be found here: https://hdl.handle.net/11600/74119.

## Supplementary material

SUPPLEMENTARY FIGURE 1 Schematic representation of the classification of glomerular tubularization: **(A)** one cross, **(B)** two crosses and **(C)** three crosses. Adapted from Silva et al. (2025).

SUPPLEMENTARY FIGURE 2 PCA biplot showing each individual subject as a point, color-coded according to the experimental group. The ellipses represent the 95% confidence regions for each group and bigger points represent the center point of each cluster. The black arrows represent the original variables projected into the PCA space. The biplot reveals a partial separation between the experimental groups. The sedentary-oil group (blue) displays greater dispersion, indicating high intra-group variability. Some individuals in this group are positioned along vectors corresponding to variables such as body weight, kidney weight, potassium excretion, and PCNA expression. In contrast, the exercise-oil group (green) is located closer to vectors associated with renal function and acid-base balance—such as bicarbonate, PCO2, hematocrit, and urinary flow—suggesting a distinct physiological profile, potentially influenced by physical activity. The exercise-hormone (red) and sedentary-hormone (purple) groups show partial overlap, but diverge along axes representing renal structural and functional variables, including glomerular tubulization, plasma creatinine, and PCNA. Overall, the PCA confirms that the combined effects of exercise and hormonal treatment result in distinct multivariate profiles, with a certain degree of group separation and clustering, as well as highlighting key variables that appear to drive these differences. This multivariate approach complements the univariate analyses and enhances the interpretation of systemic effects induced by the different experimental conditions.

SUPPLEMENTARY FIGURE 3 PCA clustering with K-means method with unsupervised 4 clusters. The bigger shape shows the center of each cluster. The *k*-means clustering analysis, applied to the PCA scores, revealed grouping patterns consistent with the experimental treatments. The blue cluster was predominantly composed of individuals from the FSH group (sedentary females treated with hormone), suggesting a relatively homogeneous morphofunctional profile within this subgroup. Similarly, the purple cluster mainly included individuals from the FEO group (females subjected to exercise and treated with oil), indicating a potential effect of physical activity on cluster formation. In contrast, the green cluster showed greater heterogeneity, comprising individuals from various experimental groups—possibly reflecting intermediate or overlapping physiological profiles, as also observed in Supplementary Figure 2. The red cluster, in turn, was smaller in size and predominantly composed of sedentary individuals treated with either oil or hormone, further supporting the influence of treatment modality on sample stratification. Overall, this analysis supports the presence of treatment-related variation in multivariate profiles across the experimental groups.
