# Supplementary figures and images for: Assessing the impact of resistance training on renal function of female wistar rats under cross-sex hormone therapy
Source: Front Physiol. 2025 May 26;16:1543077. doi: 10.3389/fphys.2025.1543077 (PMC12146172; doi:10.3389/fphys.2025.1543077)

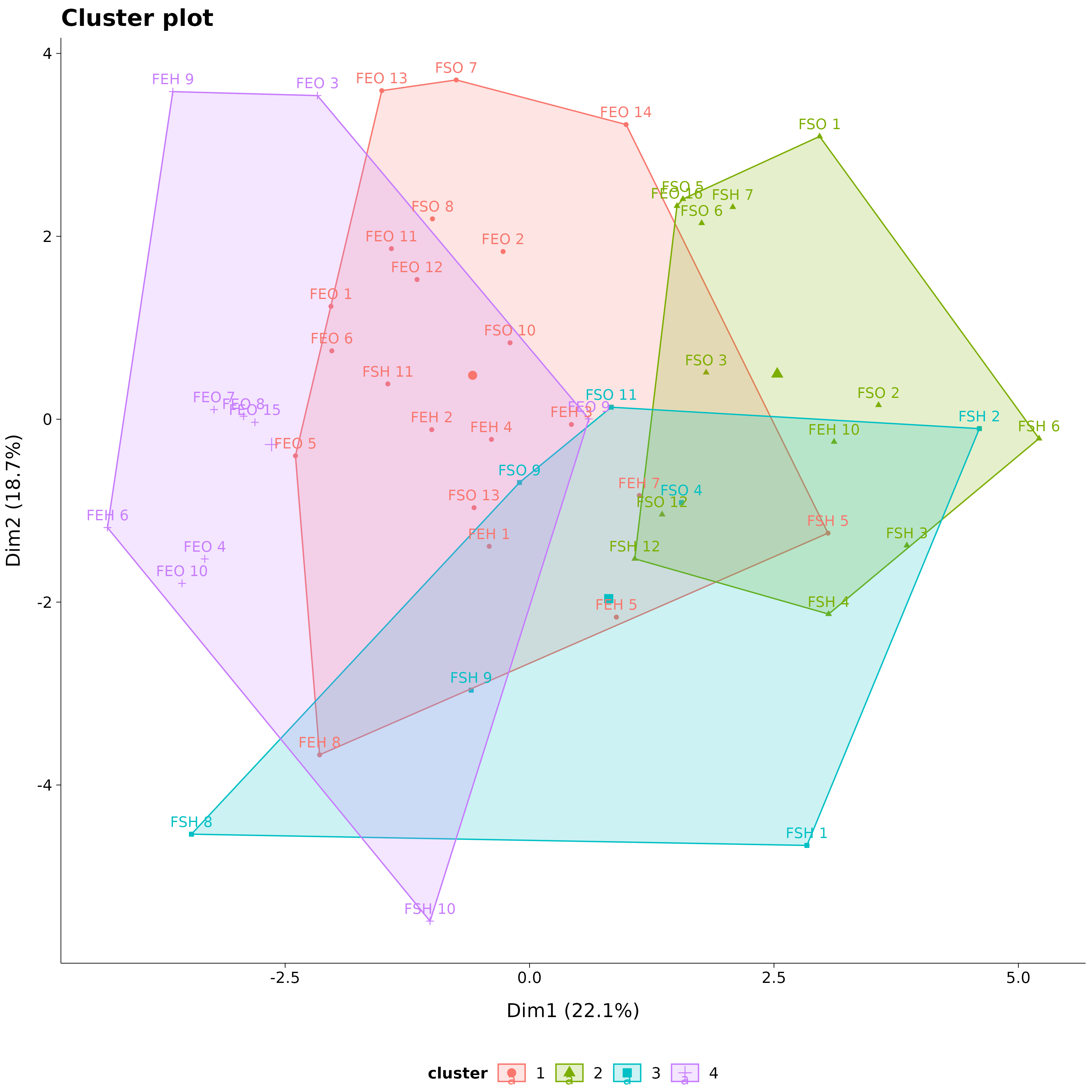

Supplement: Supplementary file 1 [file Image3.jpeg]

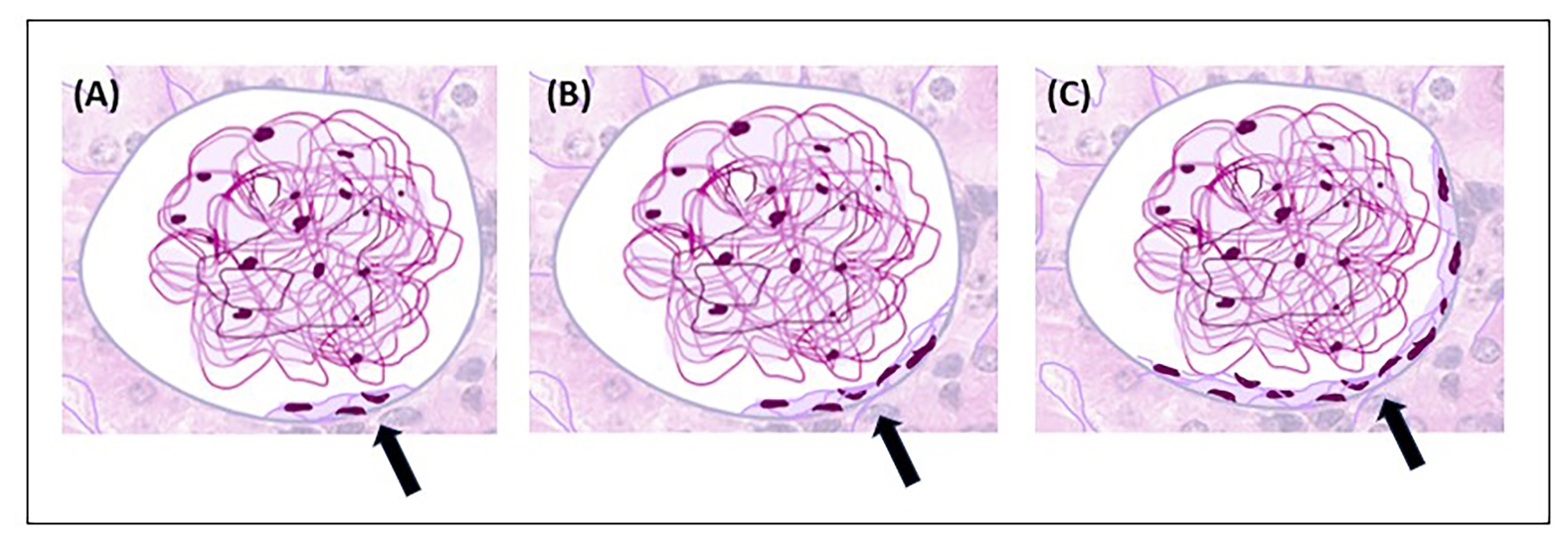

Supplement: Supplementary file 2 [file Image1.jpeg]

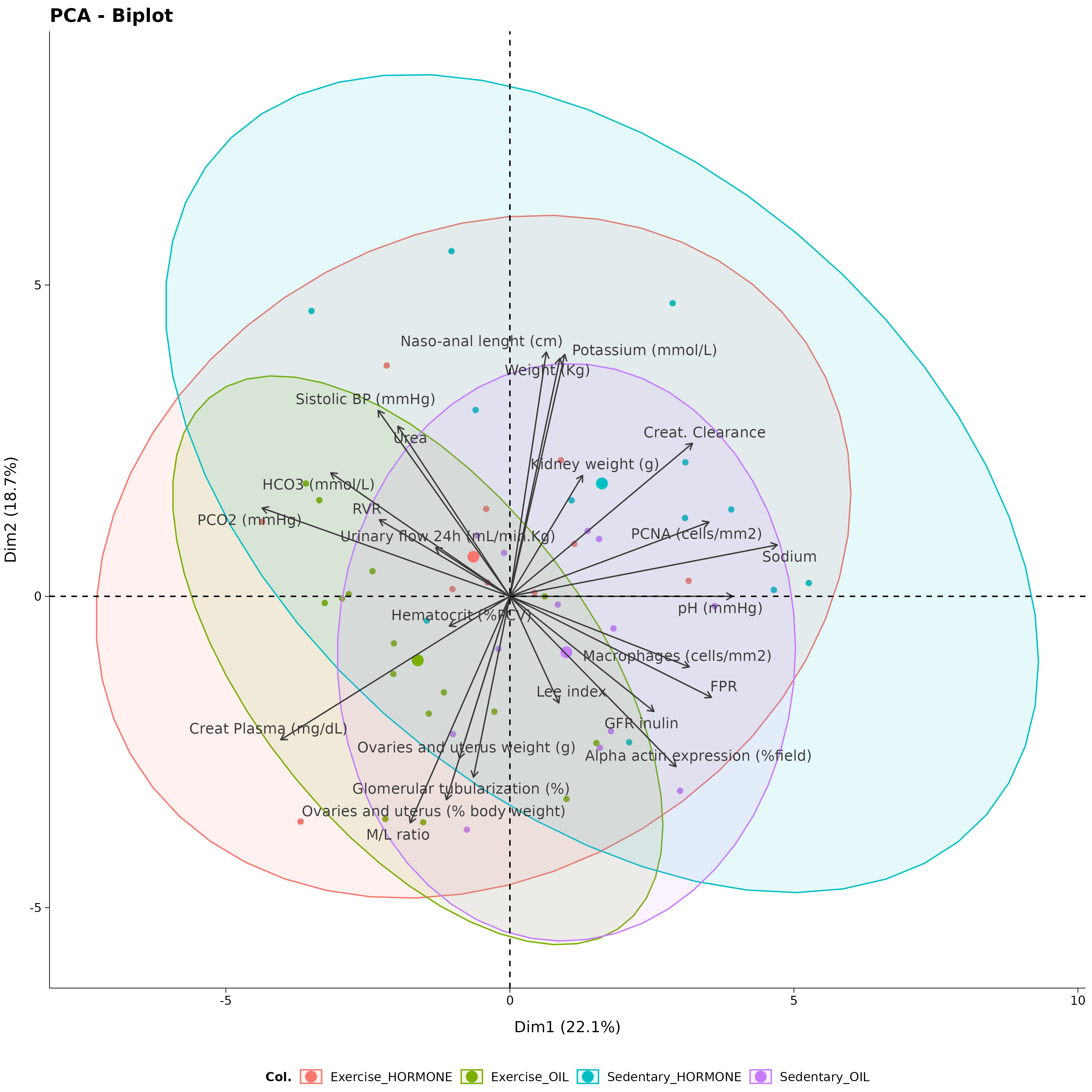

Supplement: Supplementary file 3 [file Image2.jpeg]
